# Feasibility of Administering an Electronic Version of the National Youth Tobacco Survey in a Classroom Setting

**DOI:** 10.5888/pcd17.190294

**Published:** 2020-02-27

**Authors:** S. Sean Hu, Andrea Gentzke, Ahmed Jamal, David Homa, Linda Neff

**Affiliations:** 1 Office on Smoking and Health, National Center for Chronic Disease Prevention and Health Promotion, Centers for Disease Control and Prevention, Atlanta, Georgia

## Abstract

**Introduction:**

The National Youth Tobacco Survey (NYTS) has successfully monitored tobacco product use patterns and correlates since 1999 among US students in grades 6 through 12 using a scannable paper-and-pencil format. We conducted a study to determine the feasibility and potential benefits of administering an electronic version of the NYTS in school settings.

**Methods:**

The pilot survey was administered by using 2 versions. Version 1 mimicked the scannable paper-and-pencil format with respect to design, formatting, and structure, but was administered on a tablet computer. Version 2 used an electronic survey design and formatting capabilities, which included programmed logic skips and tobacco product images. Chi-square and *t* tests were used to assess subgroup differences. Multivariable-adjusted logistic regression models were used to determine if the odds of ever and current tobacco product use differed between the 2 versions.

**Results:**

In total, 2,769 students completed version 1 or version 2. Three-quarters of respondents reported a strong preference for using an electronic device to take the NYTS (74.7%). Compared with version 1, version 2 reduced the mean time to complete the survey by 15% (*P* < .01), reduced the number of questions students needed to answer by 30% (*P* < .01), and removed 1.9% of inconsistent survey responses. A significant difference was observed for ever e-cigarette use between versions 1 (22.2%) and 2 (29.5%; *P* < .0001). No significant differences in ever or current use were observed for other tobacco products.

**Conclusion:**

An electronic mode of administration is feasible and valid for conducting surveillance of tobacco product use among US youths.

**Summary**

**What is already known on this topic?**

Multiple studies have identified advantages of electronic survey administration (eg, using tablet computers with programmed logic skips and tobacco product images) over paper-and-pencil administration, including a reduction in logic inconsistences and item nonresponse, improved respondent recall, and better efficiency of data cleaning.

**What is added by this report?**

Administering the National Youth Tobacco Survey (NYTS) electronically in school classroom settings was feasible, was well accepted by respondents, and improved efficiency of survey administration.

**What are the implications for public health practice?**

Electronic administration of the NYTS reduces respondent burden and can lead to more timely and valid surveillance of tobacco product use among youths.

## Introduction

To reduce tobacco-related death, disease, and disability, timely data are needed to inform evidence-based tobacco use prevention and control strategies (1). Such data are particularly important given that the range of tobacco products available on the US market has expanded in recent years and continues to evolve at a rapid pace. Current national tobacco surveillance systems have been successful in monitoring tobacco product use patterns and correlates for several decades (2,3); however, modernization of these surveillance systems is important to ensure the timely collection and dissemination of data that are germane to the diversified tobacco landscape in the United States (1).

Advances in information technology and wireless connectivity have bolstered the technical and fiscal feasibility of electronic (mobile digital device) survey administration in various settings, such as classrooms. Furthermore, current technology allows for the collection of data without having to rely on the internet, Wi-Fi capabilities, or information technology infrastructure of the survey setting. Multiple studies have identified advantages of electronic data collection (4–7), including the ability to embed logic skips, incorporate program checks to mitigate logic inconsistencies, add prompts to reduce item nonresponse, add images to help with recall, analyze paradata (data about the survey process) to detect potential issues with item performance, allow real-time monitoring of survey completion, and increase efficiency of data cleaning, leading to earlier release of data sets for analyses.

The National Youth Tobacco Survey (NYTS) is administered to US middle and high school students (grades 6–12) to collect data on tobacco product use behaviors, knowledge, and attitudes, exposure to secondhand emissions from tobacco products, exposure to protobacco and antitobacco influences, and other correlates of tobacco product use. The NYTS data are used to monitor trends in tobacco product use, identify sociodemographic characteristics as correlates to tobacco use behaviors, and determine the factors that either promote or discourage tobacco use (8). Since its inception in 1999, the NYTS has been administered via a paper-and-pencil scannable form; NYTS was conducted usually every 2 to 3 years through 2011 and has been conducted annually since then.

In 2018, CDC conducted a pilot study to determine the feasibility and potential benefits of administering an electronic version of NYTS, which included programmed logic skips and tobacco product images. The purpose of this study was to assess 1) respondent burden, 2) any efficiencies in using logic skips, 3) data validation and turnaround time in the dissemination of results, and 4) students’ perceptions of confidentiality when using an electronic mode of collection.

## Methods

### NYTS design

The NYTS electronic pilot was administered in parallel with the full-scale annual paper-and-pencil NYTS during March through May 2018. The pilot and traditional NYTS were conducted by using separate independent samples of students. A stratified, 3-stage cluster sample design was used to produce a nationally representative sample. Sampling procedures were probabilistic, conducted without replacement at all stages, and entailed selection of 1) primary sampling units (PSUs) (defined as a county, or a group of small counties, or part of a very large county) within each stratum, 2) secondary sampling units (defined as schools or linked schools) within each selected PSU, and 3) students within each selected school. Participation in the NYTS is voluntary at both the school and student levels. Parental consent and youth assent are required for participation in the NYTS. Further information on sample design, procedures of school recruitment and class selections, and data weighting is available at https://www.cdc.gov/tobacco/data_statistics/surveys/nyts/index.htm.

### Electronic pilot survey

The NYTS electronic pilot survey was administered as a self-interview by using a mobile digital device (Apple iPad, Apple, Inc) among a sample of middle and high school students. The sample design, school recruitment, and class selection procedures of the pilot survey were identical to the paper-and-pencil NYTS. The electronic data collection was conducted without the use of any school-based information technology infrastructure, such as a computer laboratory or Wi-Fi internet connectivity. For the pilot administration, 160 mobile digital devices were preprogrammed using Qualtrics survey software (Qualtrics), which provided the student access to the survey instrument without depending on an active internet connection. Participating students completed the pilot survey in person in a classroom setting on the provided mobile digital devices. Data collectors transported the mobile digital devices between locations in durable travel cases. The final sample comprised 36 of the 60 sampled schools (school participation rate: 60.0%), which yielded 2,769 completed interviews from the 3,398 sampled students (student participation rate: 81.5%). The overall participation rate for the pilot survey was 48.9%.

To assess the impact of programmed logic skips and the inclusion of tobacco product images in an electronic survey administration, this pilot was administered under 2 survey conditions: a version without tobacco product images and logic skips (version 1) and a version with tobacco product images and programmed logic skips (version 2). Pilot version 1 was designed to be near-identical to the scannable paper-based NYTS in respect to design, formatting, and structure. Version 2 was designed to leverage the electronic survey design and formatting capabilities by modifying the look and feel of the pilot survey for an electronic mode of administration. For example, a graphical user interface was used that included elements such as colors, shapes, images, layout, and typefaces (the “look”) as well as various behavioral elements such as response buttons, graphical controls, and survey flow (the “feel”). Students in each selected classroom were assigned randomly to participate in 1 of the 2 pilot versions. The time to complete the survey was recorded for each participant by using an internal timer in the survey application. The time stamp started at the point of opening the first question and stopped at the point of submitting the survey.

### Measures

Participants were asked about ever use and current use of cigarettes, cigars, smokeless tobacco, electronic cigarettes (e-cigarettes), hookahs (waterpipes), pipe tobacco, snus, dissolvable tobacco products, bidis, and roll-your-own cigarettes. For each product, ever use was defined as ever having tried or used, even on 1 or 2 occasions. Current use was defined as use on 1 or more days during the past 30 days.

Five questions were added to both pilot survey versions to assess students’ perceptions and previous use of electronic devices for digital-based data collections. These were 1) “If you had a choice of taking this survey using an electronic device or taking this survey using paper and pencil, which would you choose?” [responses: electronic device, paper and pencil, not sure]; 2) “How much do you agree or disagree with the statement that using an electronic device to take this survey made you feel nervous?” [responses: strongly agree, agree, neither agree nor disagree, disagree, strongly disagree]; 3) “How much do you agree or disagree that using an electronic device keeps this survey from being private?” [responses: strongly agree, agree, neither agree nor disagree, disagree, strongly disagree]; 4) “How often do you use an electronic device at school, home, or work? Include activities such as being on the Internet, computer games, and e-mail.” [responses: multiple times per day, once per day, multiple times per week, once per week, multiple times per month, once per month, yearly or less, I do not use electronic devices]; and 5) “(Before today), have you ever used an electronic device to take a survey or test?” [responses: yes, no, not sure].

The presence of contradictory, inconsistent, or illogical responses was determined by applying a series of data cleaning edit check rules across both survey versions after finishing data collection. The edit check rules applied logic to students’ responses to 53 tobacco product use behavior questions to determine if the answers provided to these specific questions were complementary, consistent, and logical, based on their responses to related screening questions. For example, if a respondent answered “no” to the question, “Have you ever tried cigarette smoking, even one or two puffs?” it should be expected that the respondent would answer “I have never smoked cigarettes, not even one or two puffs” to the subsequent questions pertaining to their experience with cigarette smoking.

### Analysis

Pilot participants came from the same sample and experienced the same data collection procedures; the only difference was the survey version. Therefore, this analysis focused on differences between the 2 pilot survey versions. Chi-square tests were conducted to assess differences in participants’ demographic characteristics, perceptions about electronic data collection, and the prevalence of ever and current use of each tobacco product between the 2 pilot survey versions. Analyses of variance were conducted to assess differences in survey completion time between versions, controlling for sex, race/ethnicity, and school grade; a Bonferroni corrected/adjusted *P* value was used to protect from type I error. Logistic regression models were used to examine the effect of survey version (version 2 vs version 1) on report of ever and current use of each tobacco product, controlling for sex, race/ethnicity, and school grade. All analyses were conducted by using SAS, version 9.4 (SAS Institute, Inc).

## Results

In total, 2,769 student respondents completed the pilot survey; 1,378 (49.8%) completed version 1 and 1,391 (50.2%) completed version 2. Pilot survey respondents were not significantly different in sex, race/ethnicity, or school grade composition by version (Table 1). Most respondents (>85%) were 12 to 17 years old.

**Table 1:** Demographic Characteristics by Survey Version, Electronic Pilot, National Youth Tobacco Survey, 2018

Characteristic	Version 1^a^	Version 2^b^	χ^2^ *P* value
	No. (%)	No. (%)	
**Total**	1,378 (100.0)	1,391 (100.0)	NA
**Sex**			
Female	636 (46.2)	647 (46.5)	.92
Male	736 (53.4)	743 (53.4)	
Unknown	6 (0.4)	1 (0.1)	
**Race/ethnicity**			
White, non-Hispanic	466 (33.8)	476 (34.2)	.45
Black, non-Hispanic	299 (21.7)	297 (21.4)	
Hispanic	405 (29.4)	406 (29.2)	
Other	156 (11.3)	189 (13.6)	
Unknown	52 (3.8)	23 (1.7)	
**School grade**			
Grade 6	243 (17.6)	242 (17.4)	>.99
Grade 7	246 (17.9)	246 (17.7)	
Grade 8	201 (14.6)	210 (15.1)	
Grade 9	190 (13.8)	201 (14.5)	
Grade 10	197 (14.3)	199 (14.3)	
Grade 11	174 (12.6)	166 (11.9)	
Grade 12	125 (9.1)	124 (8.9)	
Unknown	2 (0.1)	3 (0.2)	

Abbreviation: NA, not applicable.

a Survey version without logic skips and images.

b Survey version with logic skips and images.

**Figure 1:**
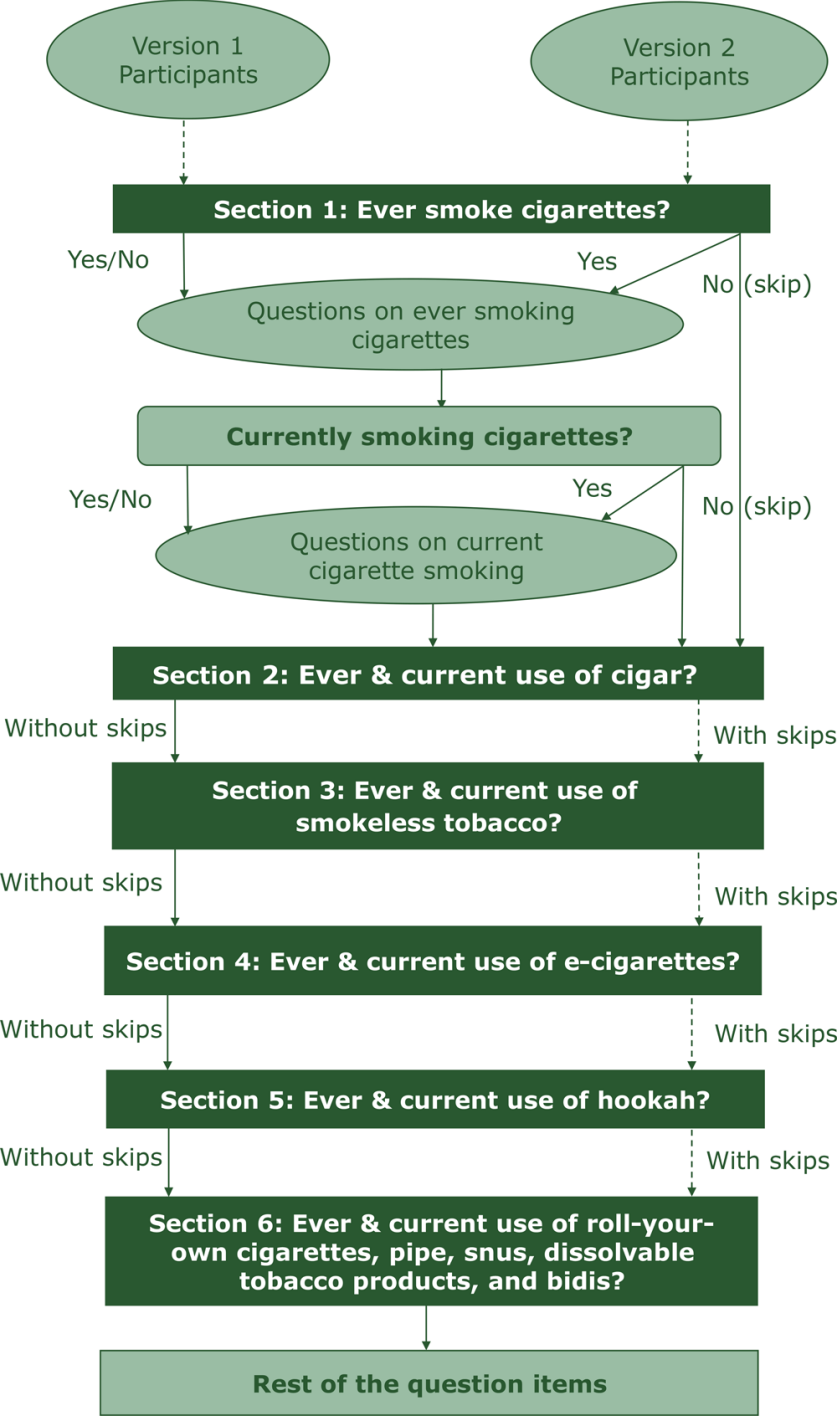
Participants response, routes by survey version in an electronic pilot, National Youth Tobacco Survey, 2018.

Respondents assigned to version 1 (without images or logic skips) were asked to read and respond to all survey questions (N = 93), regardless of their tobacco product use behaviors, similar to the paper-and-pencil NYTS questionnaire (Figure 1; Table 2). However, respondents assigned to version 2 (with images and logic skips) responded to between 59 questions (never user of tobacco product) and 93 questions (current user of ≥1 products), depending on their tobacco use behaviors. Version 2 respondents were asked an average range of 64 to 66 survey items, or approximately 30% fewer questions than in version 1.

**Table 2:** Respondent Survey Burdens Stratified by Tobacco Use Behavior and Survey Version, Electronic Pilot, National Youth Tobacco Survey, 2018

Behavior	Mean Time to Complete the Survey^a^					No. of Questions Asked^b^	
	Version 1 (n = 1,378)		Version 2 (n = 1,391)		*P* Value	Version 1	Version 2, Range
	n (%)	Minutes	n (%)	Minutes	(Version 1 vs Version 2)^c^		
**Overall**	1,214 (100.0)	13.5	1,217 (100.0)	11.5	<.0001	93	64–66^d^
**Tobacco Use Behavior**							
**Never use of any tobacco product**	792 (65.2)	13.8	746 (61.3)	11.4	<.0001	93	59
**Ever use^e^**							
1 product used	133 (11.0)	13.1	143 (11.8)	10.4	<.0001	93	67–69
2 products used	53 (4.4)	13.7	67 (5.5)	11.0	<.0001	93	69–72
≥3 products used	34 (2.8)	13.2	42 (3.5)	12.9	.58	93	71–81
**Current use^f^**							
1 product used	117 (9.6)	12.9	121 (9.9)	12.0	.0057	93	72–78
2 products used	38 (3.1)	12.6	56 (4.6)	12.6	.91	93	74–84
≥3 products used	47 (3.9)	12.3	42 (3.5)	13.3	.25	93	77–93
**Current Use, by Product^g^**							
**E-cigarettes**							
Yes	123 (10.2)	12.5	145 (12.0)	12.4	.81	93	77–93
No	1,079 (89.8)	13.7	1,068 (88.0)	11.3	<.0001	93	59–92
**Cigarettes**							
Yes	52 (4.3)	13.1	47 (3.9)	13.7	.47	93	78–93
No	1,151 (95.7)	13.5	1,165 (96.1)	11.4	<.0001	93	59–89
**Cigars**							
Yes	77 (6.5)	13.0	90 (7.4)	12.3	.25	93	74–93
No	1,112 (93.5)	13.6	1,125 (92.6)	11.4	<.0001	93	59–92
**Smokeless tobacco**							
Yes	25 (2.1)	12.1	29 (2.4)	13.3	.17	93	73–93
No	1,172 (97.9)	13.6	1,185 (97.6)	11.4	<.0001	93	59–93
**Hookah**							
Yes	39 (3.2)	12.3	27 (2.2)	11.8	.61	93	74–93
No	1,163 (96.8)	13.6	1,188 (97.8)	11.5	<.0001	93	59–92
**Any product use**							
Yes	196 (16.2)	12.7	253 (20.2)	12.4	.27	93	72–93
No	1,015 (83.8)	13.7	1,002 (79.8)	11.3	<.0001	93	59–83

a Analysis based on 2,431 respondents; 333 missing cases due to missing survey burden data and 5 outliers were not included in the survey burden analysis.

b For version 1 that did not contain any logic skips or images, all respondents were asked all 93 survey questions. For version 2 that was programmed with logic skips based on respondents’ self-reported tobacco product use behaviors, respondents skipped inapplicable questions based on their tobacco product use status. A range of questions was reported, when applicable, providing the lowest and highest number of questions that would be asked based on varying tobacco product use behaviors (eg, range from never user of all other products to current user of all products).

c Time to complete (minutes) compared by analysis of variance, controlling for sex, race/ethnicity, and school grade. Because we conducted 20 multiple analyses on the same dependent variable (time to complete), a Bonferroni correction was conducted for protecting from type I error. The Bonferroni corrected/adjusted *P* value: .05/20 = .0025. Therefore, significant differences were *P* < .0025.

d The overall range was the sum of the number of products students could report having used and the number of questions needed to be completed for each group of tobacco product use behaviors (eg, (61.3% × 59 + 11.8% × 67 + 5.5% × 69 + 3.5% × 71 + 9.9% × 72 + 4.6% × 74 + 3.5% × 77) = 64).

e Defined as ever use of any of the following tobacco products, even just 1 time in the entire life but not use of them in the past 30 days: cigarettes, cigars, smokeless tobacco, e-cigarettes, hookah, pipe tobacco, roll-your-own cigarettes, snus, dissolvable tobacco products, and bidis.

f Defined as use on at least 1 day during the past 30 days of any of the following tobacco products: cigarettes, cigars, smokeless tobacco, e-cigarettes, hookah, pipe tobacco, roll-your-own cigarettes, snus, dissolvable tobacco products, and bidis.

g Defined as reported use on at least 1 day during the past 30 days for each listed tobacco product (with or without use of any other product). Numbers may not equal totals because of missing data.

The mean completion time for respondents to version 1 was 13.5 minutes, which was significantly higher than the mean completion time for respondents to version 2 (11.5 minutes; *P* < .0001), which included programmed logic skips (Table 2). No significant difference in completion time was observed by version among respondents reporting ever use of 3 or more tobacco products or current use of 1 or more tobacco products. No significant differences in completion time were observed among those reporting current use of any tobacco product.

The survey included 53 questions about tobacco product use behaviors that were subject to potential contradictory, inconsistent, or illogical responses. Among all version 1 (no images or logic skips) participants (n = 1,378), there were 73,034 total question items that were potentially subject to manual edit check corrections. Overall, 1,413 of these 73,034 items (1.9%) were discrepant on this edit check and required manual correction (responses set to missing). Comparatively, no survey items required edit check corrections for version 2, which used programmed logic skips. Although race/ethnicity had the highest individual item nonresponse rates for both versions (version 1, 6.9%; version 2, 3.3%), version 2 yielded fewer respondents with missing data (item nonresponse rate was <1% for all other nonskip and current tobacco use survey items; range 0%–0.9%) compared with version 1 (item nonresponse rates were >1% for 60% of all other nonskip and current tobacco use questions; range, 0.2%–2.7%). Between the 2 survey versions, there was a significant difference in response rates for 88% of the questions (*P* < .05).

A greater proportion of version 2 respondents reported ever use of e-cigarettes compared with version 1 respondents (29.5% vs 22.2%, *P* < .01). After adjusting for sex, race/ethnicity, and grade level, the odds of reporting ever e-cigarette use was 1.5 times higher for version 2 respondents compared with version 1 (OR, 1.5; 95% CI, 1.3–1.8). No other significant differences in tobacco product use (ever or current) were observed by version (Table 3).

**Table 3:** Prevalence and Adjusted Odds Ratios for Each Key Tobacco Product Use Behavior^a^ by Survey Version^b^, Electronic Pilot, National Youth Tobacco Survey (NYTS), 2018

Tobacco Products	Version 1, %	Version 2, %	Adjusted Odds Ratio^c^	*P* Value
**Current use**				
Cigarettes	4.5	3.8	0.8	0.26
Cigars	5.8	6.8	1.2	0.35
Smokeless tobacco	2.3	2.3	0.9	0.82
E-cigarettes	11.8	13.1	1.1	0.34
Hookah	2.9	2.2	0.7	0.25
Pipe tobacco	1.0	0.7	0.6	0.24
Snus	1.6	1.3	0.8	0.43
Bidis	0.8	0.5	0.6	0.30
Roll-your-own cigarettes	1.7	1.7	0.9	0.77
Any tobacco^d^	17.7	18.8	1.1	0.35
**Ever use**				
Cigarettes	17.0	16.5	1.0	0.72
Cigars	15.6	17.8	1.2	0.12
Smokeless tobacco	6.7	5.9	0.9	0.40
E-cigarettes	22.2	29.5	1.5	<.0001
Hookah	7.8	7.6	1.0	0.88
Pipe tobacco	2.8	2.5	0.9	0.57
Snus	4.3	3.8	0.9	0.65
Bidis	1.9	1.9	1.0	0.93
Roll-your-own cigarettes	4.1	4.6	1.1	0.50
Any tobacco^e^	35.8	38.6	1.2	0.06

a Prevalence estimates for dissolvable tobacco products not presented because of small sample size (n < 10).

b Version 1: survey version without logic skips and images; version 2: survey version with logic skips and images.

c Adjusted odds ratio for version 2 versus version 1 (reference group). In logistic regression models, results were adjusted for gender, race/ethnicity, and school grade.

d Current use of any tobacco product was defined as use of 1 or more tobacco product (cigarette, cigars, smokeless tobacco, e-cigarettes, hookah, pipe tobacco, roll-your-own cigarettes, dissolvable tobacco products, snus, and bidis) on at least 1 day in the past 30 days.

e Ever use of any tobacco product was defined as ever use, even just 1 time, of 1 or more tobacco product (cigarettes, cigars, smokeless tobacco, e-cigarettes, hookah, pipe tobacco, roll-your-own cigarettes, dissolvable tobacco products, snus, and bidis) in the entire life.

**Figure 2:**
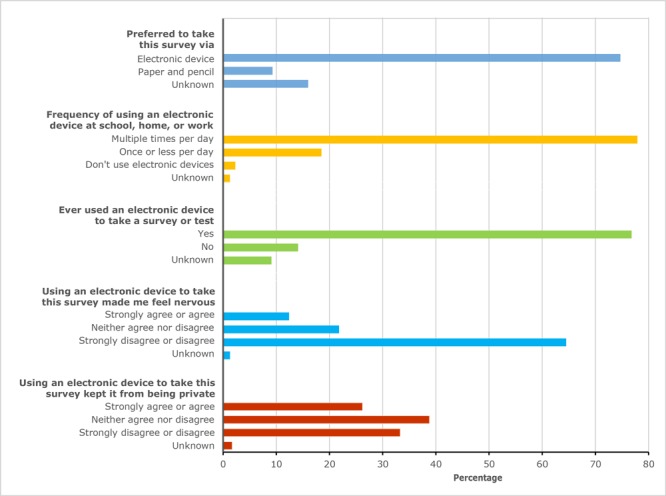
Participant attitudes toward an electronic survey, electronic pilot, National Youth Tobacco Survey (NYTS), 2018.

Among all pilot survey respondents (N = 2,769), most (74.7%) reported a preference for using an electronic device to respond to the survey or were unsure of their preference (16.0%); only 9.3% preferred a paper and pencil survey (Figure 2). Most of students (64.5%) also strongly disagreed or disagreed with the statement, “Using an electronic device to take this survey made me feel nervous”; 12.4% strongly agreed or agreed with this statement, while 21.8% said they neither agreed nor disagreed. Perceptions of privacy with using an electronic device were high; 26.2% of respondents strongly agreed or agreed that “Using an electronic device keeps this survey from being private.” Furthermore, most participants (77.9%) reported using an electronic device at school, home, or work multiple times per day; only 2.3% said they did not use electronic devices at all. Finally, most students (76.8%) said that they have previously taken a survey or test on an electronic device.

**Table:** 

Category/response	%
**Preferred to take this survey via**	
Electronic device	74.7
Paper and pencil	9.3
Unknown	16.0
**Frequency of using an electronic device at school, home, or work**	
Multiple times per day	77.9
Once or less per day	18.5
Don’t use electronic devices	2.3
Unknown	1.3
**Ever used an electronic device to take a survey or test**	
Yes	76.8
No	14.1
Unknown	9.1
**Using an electronic device to take this survey made me feel nervous**	
Strongly agree or agree	12.4
Neither agree nor disagree	21.8
Strongly disagree or disagree	64.5
Unknown	1.3
**Using an electronic device to take this survey kept it from being private**	
Strongly agree or agree	26.2
Neither agree nor disagree	38.8
Strongly disagree or disagree	33.3
Unknown	1.7

## Discussion

Although the use of combustible tobacco products among middle and high school students has declined since 2011, the overall use of any tobacco product has increased since 2015 (9), and the US Surgeon General has declared youth use of e-cigarettes to be an epidemic (10). Surveillance of tobacco product use behaviors is critical to obtain data to inform tobacco control policy and practice. The NYTS has been conducted since 1999 by using a scannable paper-and-pencil format and has successfully monitored tobacco product use patterns and correlates; however, the time between data collection and dissemination of findings could be shortened significantly by modernizing the survey to an electronic version. Reducing the dissemination time is important, given the rapidly changing tobacco landscape, particularly among youths.

These findings support the use of an electronic mode of administration to survey US middle and high school students in a classroom setting. The electronic survey administration was well accepted; most respondents reported familiarity with using electronic devices in their daily lives; and over three-quarters had previously taken an electronic survey or test. Furthermore, reported concerns about privacy while using an electronic device did not apparently translate to reluctance to take the survey. These results are consistent with previous findings that digital-based surveys significantly increased responses to sensitive questions (11–13) and are more accepted than a paper-based survey (14). Thus, these findings indicate that an electronic mode of administration to assess tobacco product use behaviors was well received among students and did not raise privacy concerns from most respondents.

Version 2 of the electronic survey, which included images and logic skips, further resulted in efficiencies of data collection. Specifically, the incorporation of logic skips and conditional routing provided a significant advantage in reducing individual respondent burden by resulting in a significantly lower average survey completion time. These reductions were primarily limited to never tobacco product users and noncurrent tobacco product users, who make up most NYTS participants. Conditional routing also resulted in a reduction in contradictory, inconsistent, and illogical survey responses and reduced item nonresponse. These benefits, in turn, may lead to an increase in overall statistical power (ie, a higher likelihood that the survey would detect an effect when an effect actually exists) and improvements in the quality of the data. As a result, the time it takes to process and disseminate the data and findings can be shortened significantly.

Consistent with the findings on tobacco product use behaviors in our pilot study, previous literature has shown that, regardless of the mode of administration, risk behavior prevalence estimates are similar (6,14). However, because of its design flexibility, an electronic survey allows for the inclusion of images, which may help survey respondents to better comprehend the questions (ie, by seeing the type of tobacco products) and thus improve their validity. Given the particularly rapid evolution of e-cigarettes in recent years (2), including the introduction of new devices resembling USB flash drives (eg, Juul), the addition of images to version 2 of the pilot survey may have contributed to the higher number of participants reporting ever e-cigarette use. Thus, the inclusion of tobacco product images to the electronic NYTS questionnaire could help students correctly identify various products, including new or evolving product types, thereby reducing recall errors. This is consistent with a previous finding that digital surveys, particularly those programmed with images and logic skips, can improve real-time consistency in responses and significantly increase the rate of affirmative responses to questions about sensitive issues (11–13).

About one-quarter of respondents reported potential privacy concerns with taking this survey electronically. To alleviate these potential concerns, data collectors told students that no identifying information, including their name, classroom, or school, was collected; data were not shared back to any individual school; and students could choose not to participate in the survey or skip any question that they did not feel comfortable answering. Although several factors may influence students’ survey completion time, such as reading speed or comprehension, in future NYTS administrations, students who finish the survey early may be asked to remain at their own seats and do homework or read while other students continue to do the survey. Thus, all students will turn in their tablets at the same time, minimizing the likelihood that they may discern their peers’ tobacco use behaviors based on differences in survey completion times.

The findings in this study are subject to limitations. First, these data are self-reported and may be subject to reporting bias. Second, the sample size for the pilot survey was small, potentially limiting the generalizability of these findings. Third, contrary to the full-scale NYTS survey, the pilot did not have a procedure for obtaining make-up surveys for students who were absent on the day of data collection. Finally, even though 81.5% of eligible students participated in the survey, the overall response rate was 48.9%, which could lead to potential bias if there are systematic differences between respondents and nonrespondents at both the school and student levels.

The results of this pilot study support using an electronic mode to conduct the annual NYTS. Electronic administration of the NYTS reduces respondent burden and can lead to more timely and valid surveillance of tobacco product use by youths.
